# Responses of Rhizosphere Fungal Communities to the Sewage Sludge Application into the Soil

**DOI:** 10.3390/microorganisms7110505

**Published:** 2019-10-29

**Authors:** Katarína Ondreičková, Marcela Gubišová, Michaela Piliarová, Miroslav Horník, Pavel Matušinský, Jozef Gubiš, Lenka Klčová, Martina Hudcovicová, Ján Kraic

**Affiliations:** 1 National Agricultural and Food Centre—Research Institute of Plant Production, Bratislavská cesta 122, 921 68 Piešťany, Slovakia; marcela.gubisova@nppc.sk (M.G.); jozef.gubis@nppc.sk (J.G.); lenka.klcova@nppc.sk (L.K.); martina.hudcovicova@nppc.sk (M.H.); jan.kraic@nppc.sk (J.K.); 2 University of Ss. Cyril and Methodius, Faculty of Natural Sciences, Námestie J. Herdu 2, 917 01 Trnava, Slovakia; misa.piliarova@gmail.com (M.P.); miroslav.hornik@ucm.sk (M.H.); 3 Department of Botany, Faculty of Science, Palacký University in Olomouc, Šlechtitelů 27, 783 71 Olomouc, Czech Republic; pavel.matusinsky@upol.cz; 4 Agrotest fyto, Ltd., Havlíčkova 2787, 767 01 Kroměříž, Czech Republic

**Keywords:** arbuscular mycorrhizal fungi, fungal community, genetic diversity, sewage sludge, T-RFLP, 18S rDNA sequencing

## Abstract

Due to the increasing sewage sludge production in the world and problems with its disposal, an application of sludge to the soil appears to be a suitable solution considering its fertilizer properties and ability to improve the soil physical conditions. On the other hand, the sludge may also contain undesirable and toxic substances. Since soil microorganisms are sensitive to environmental changes, they can be used as indicators of soil quality. In this study, we used sewage sludge (SS) from two municipal wastewater treatment plants (SS-A and SS-B) in the dose of 5 t/ha and 15 t/ha in order to determine possible changes in the fungal community diversity, especially arbuscular mycorrhizal fungi (AMF), in the rhizosphere of *Arundo donax* L. Rhizosphere samples were collected in summer and autumn for two consecutive years and the fungal diversity was examined using terminal restriction fragment length polymorphism and 18S rDNA sequencing. Fungal alpha diversity was more affected by SS-A than SS-B probably due to the higher heavy metal content. However, based on principal component analysis and ANOSIM, significant changes in overall fungal diversity were not observed. Simultaneously, 18S rDNA sequencing showed that more various fungal taxa were detected in the sample with sewage sludge than in the control. *Glomus* sp. as a representative of AMF was the most represented. Moreover, *Funneliformis* in both samples and *Rhizophagus* in control with *Septoglomus* in the sludge sample were other representatives of AMF. Our results indicate that the short-term sewage sludge application into the soil does not cause a shift in the fungal community composition.

## 1. Introduction

Sewage sludge is a byproduct of the wastewater treatment process, and its production in the world continues to grow. Disposal of sludge in the European Union (EU) is carried out in several ways—agricultural use, compost, landfill, dumping at sea, incineration, and other applications [1]. Since sludge contains macro and microelements, which are an important source for plant nutrition and also a high proportion of organic matter, it would be appropriate to use it as a fertilizer in agriculture or as a soil conditioner. On the other hand, the sludge may contain heavy metals, harmful microorganisms (thermo-tolerant coliform bacteria, fecal streptococci, and others) or organic pollutants [2], and thus its use in agriculture in Europe is defined by Council Directive 86/278/EEC of 12 June 1986 on the protection of the environment, and in particular of the soil, when sewage sludge is used in agriculture; and in Slovakia by Act No. 188/2003 Z.z. These laws set out strict criteria that must be fulfilled before the sludge is applied into the soil. The application of sludge to agricultural soil in Slovakia was 0% since 2014 [1]. On the other side, available data from Eurostat indicates that in 2017 Ireland, Lithuania, and the Czech Republic applied 79%, 49%, and 46% of the sewage sludge into the agricultural soil, respectively [1].

Application of sludge into the soil affects soil’s physical, chemical, and biological properties [2] as a result of the high content of organic matter [3]. This sludge addition can change soil pH [4], decrease bulk density and erosion [5], or increase soil aggregate stability, porosity, water holding capacity, humus content, heavy metals, electrical conductance, cation exchange capacity, content of N and P, and harmful microorganisms [5,6,7]. Furthermore, an organic matter content is related to changes in soil microbial communities [8,9] and application of sewage sludge in recommended amounts increases microbial activities [10]. Soil microorganisms play a crucial role in various biogeochemical cycles, also in the formation of soil structure, the decomposition of soil organic matter, and the recycling of nutrients [11], thus they are sensitive to various environmental changes and consequently can be used as indicators of soil quality [12]. Soil fungal communities are more affected by abiotic environmental factors than biotic factors [13] and their importance varies across different environments [11]. Similarly, soil fungal communities are formed by a local plant community that affords them with nutritional resources and acts as a host of certain fungal groups [14,15]. Approximately 80% of terrestrial plant species form some of the six types of mycorrhizal symbiosis, which are categorized according to clear morphological characteristics [16,17]. From them, arbuscular mycorrhiza (AM) is the most widespread and a predominant type [18]. Arbuscular mycorrhizal fungi (AMF) belong to the phylum *Glomeromycota* that colonize a wide spectrum of mono and dicotyledonous plants without the host plant specificity [19,20]. AMF have very important functions that enhance plant production or affect soil properties, and generally they affect the entire ecosystem [18]. Their significant function is also in phytoremediation of heavy metals in contaminated soil [21], and plant-AMF symbiosis or targeted plant inoculation with tolerant or stress-adapted AMF could be a potential biological solution for effective restoration of contaminated ecosystems [22,23].

In this study, we investigated the dynamics of fungal communities, especially arbuscular mycorrhizal fungi, in the rhizosphere of *Arundo donax* L. planted in the soil with the addition of sewage sludge over two years. *Arundo donax,* as a promising energy crop from family *Poaceae,* was selected because it can help to meet renewable energy targets set by the EU directive. It produces a huge amount of biomass even under low-input cultivation. Simultaneously, this plant is able to grow in contaminated or other marginal soils [24]. To increase yield of biomass, application of sewage sludge as a low-cost nutrient may be more economical than N-fertilizers. This species was also selected for the reason that it is grown for non-food purposes and the application of sewage sludge as a source of nutrients may be more acceptable there than for food crops. Therefore, we evaluate dynamics of fungal communities from the rhizosphere of *Arundo donax* planted in the soil with the sewage sludge addition in doses of 5 t/ha and 15 t/ha using terminal restriction fragment length polymorphism (T-RFLP). At the same time, we used 18S rDNA sequencing to reveal the rhizosphere fungal spectrum and possible changes in the AMF communities in the presence of sewage sludge. We assumed that the sewage sludge addition into the soil would appreciably amend fungal alpha diversity and that this would be more pronounced in the dose of 15 t/ha. Furthermore, we assumed that the sludge, as well as the seasonal effect, would have an impact on the rhizosphere fungal community composition. 

## 2. Materials and Methods

### 2.1. Study Description and Sewage Sludge Used

Plants of *Arundo donax* L. (giant reed) used in this experiment were multiplied in explant culture by the method of in vitro tillering [25]. Plantlets were transplanted to garden substrate and acclimatized to ex vitro conditions for 8 weeks. After acclimatization, the plants were transplanted to the pots (1 plant/1 pot), filled with 7 kg of arable soil supplemented with dried sewage sludge (SS) in doses of 5 or 15 t/ha, and control variant was without any supplement. Soil type used was Luvi-Haplic Chernozem on loess with pH (KCl) 6.3, medium content of humus (1.77%), low content of nitrogen (0.096%)—measured by Dumas method, good content of potassium (196 mg/kg) and phosphorus (97 mg/kg), and a high content of magnesium (280 mg/kg)—measured by Mehlich III extraction and atomic emission spectroscopy. Two different samples of SS were used: SS-A from the wastewater treatment plant Pannon-Víz Zrt.,Győr, Hungary, in the growing season 2014, and SS-B obtained from the wastewater treatment plant Tavos, a.s., Piešťany, Slovakia, in the growing season 2015. Elemental analyses of macroelements and heavy metals (these did not exceed the limits permitted by the Act No. 188/2003 in the Slovak Republic) in used SSs are shown in Table 1. Plants were cultivated from May to December in natural outdoor conditions and regularly irrigated if needed. At the end of 2014, the pot experiment with plants and sludge SS-A was discarded. In 2015, new plants were planted in pots under the same conditions as in the previous year but using sludge SS-B.

### 2.2. Rhizosphere Sampling and DNA Isolation

The samples were collected twice in 2014 (August and November) and twice in 2015 (August and December) from the rhizosphere of *Arundo donax* and each sample was taken individually from separate pots. Three pots/3 individual samples were considered as controls with arable soil only, 3 pots/3 individual samples were supplemented with sewage sludge with the dose of 5 t/ha and 3 pots/3 individual samples with the dose of 15 t/ha. The mgDNA was extracted from 0.25 g of fresh rhizosphere samples using the PowerSoil^TM^ DNA Isolation kit (Qiagen, Hilden, Germany). Extracted DNA was dissolved in 50 μL of nuclease-free water. The quantity and purity of DNA were measured spectrophotometrically with NanoDrop-1000 Spectrophotometer (Thermo Fisher Scientific Inc., Waltham, MA, USA), and samples were diluted to the same final concentration (25 ng·μL^−1^), and stored at −20 °C.

### 2.3. Terminal-Restriction Fragment Length Polymorphism

The AM fungal partial 18S rRNA gene sequences were amplified using Nested PCR with two conserved primer pairs: NS1–NS4 [26] and NS31–AM1 [27]. The first primer pair amplified PCR products of approximately 1100 bp and the second about 550 bp. Forward primer NS31 is a universal eukaryotic primer and reverse primer AM1 is a specific primer for AMF however, other studies have shown that they also amplify DNA from *Ascomycota* and *Basidiomycota* [28,29]. The second forward and reverse primers were labelled at the 5’-end with FAM and VIC fluorescent dyes, respectively. First DNA amplification with NS1–NS4 primers was carried out in 20 µL reaction mixture containing 1 × PCR buffer (Invitrogen, Thermo Fisher Scientific Inc., Waltham, MA, USA), 1.5 mM MgCl_2_ (Invitrogen, Thermo Fisher Scientific Inc., Waltham, MA, USA), 0.05 μM of each forward and reverse primer, 0.2 mM dNTP (Invitrogen, Thermo Fisher Scientific Inc., Waltham, MA, USA), 1 U of Taq DNA polymerase (Invitrogen, Thermo Fisher Scientific Inc., Waltham, MA, USA), and 25 ng of mgDNA using the GeneAmp PCR System 9700 (Applied Biosystems, Thermo Fisher Scientific Inc., Waltham, MA, USA). The PCR conditions were as follows: initial denaturation at 94 °C for 3 min, 35 cycles of denaturation at 94 °C for 30 s, annealing at 40 °C for 1 min, elongation at 72 °C for 1 min, and final elongation at 72 °C for 10 min. The samples with amplified DNA from this first PCR were diluted 1:100 and 1 µL was used in the second PCR with fluorescently labeled NS31–AM1 primers. The composition of the PCR mixture and PCR condition were the same as in the first PCR except for the annealing temperature, which was 60 °C in this case. PCR amplification was controlled electrophoretically in 1% (*w*/*v*) agarose in 1 × TBE buffer (1.1% (*w*/*v*) Tris-HCl; 0.1% (*w*/*v*) Na_2_EDTA 2H_2_O; 0.55% (*w*/*v*) boric acid) pre-stained with 0.10 µL·mL^−1^ of ethidium bromide. PCR products were purified by the PCR Purification & Agarose Gel Extraction Combo kit (Thermo Fisher Scientific Inc., Waltham, MA, USA), and dissolved in sterile water. Purified PCR products were digested with HinfI and Hsp92II restriction enzymes (Promega Corp, Madison, WI, USA) in 20 μL of digestion mixture contained 10 U of restriction enzyme, 1 × buffer, 0.1 mg·mL^−1^ of bovine serum albumin and 10 μL of purified PCR products. The mixture was incubated for 3 h at 37 °C and then purified using a purification kit and dissolved in sterile water. One microliter of purified products was mixed with 9 µL of formamide containing LIZ 600 size standard (Applied Biosystems, Thermo Fisher Scientific Inc., Waltham, MA, USA), denatured at 95 °C for 3 min, and separated by capillary electrophoresis using the ABI 3100 Prism Avant (Applied Biosystems, Thermo Fisher Scientific Inc., Waltham, MA, USA). Electropherograms were analyzed by the Peak Scanner 2 (Applied Biosystems, Thermo Fisher Scientific Inc., Waltham, MA, USA), and terminal restriction fragments (T-RFs) in range 60–550 bp were used for evaluation. Only peaks above the threshold of 50 fluorescence units were considered. The DNA from 1 sample was amplified with both labeled forward and reverse primers and subsequently, restriction digested with two enzymes, therefore, 1 sample was characterized by 4 resultant T-RFLP profiles.

### 2.4. Construction of 18S rDNA Clone Library

Fungal clone library was created from metagenomic DNA isolated from rhizosphere of *Arundo donax* without SS and with SS in doses of 15 t/ha. The partial 18S rRNA gene was amplified using Nested PCR with the conserved primer pairs as described above. PCR products were ligated into the pGEM-T Easy vector (Promega Corp., Madison, WI, USA) and transformed into competent *E. coli* TOP10F’ (Invitrogen, Thermo Fisher Scientific Inc., Waltham, MA, USA), according to the manufacturer’s instructions. Plasmid clones were identified based on blue–white screening and isolated by GeneJET Plasmid Miniprep kit (Fermentas, Thermo Fisher Scientific Inc., Waltham, MA, USA). Isolated plasmids were digested with EcoRI restriction enzyme (Promega Corp., Madison, WI, USA) to check the presence of the insert of correct size. Digestion mixture (20 μL) contained 6 U of restriction enzyme, 1 × buffer, 0.1 mg·mL^−1^ of BSA, and 5 μL of plasmid DNA. This mixture was incubated for 2 h at 37 °C and controlled electrophoretically in 1% (*w*/*v*) agarose gel. Variation of cloned inserts was assessed by T-RFLP according to the procedure described above, and clones with different T-RFLP profiles were sequenced using SP6 and T7 primers in the ABI 3100 Prism Avant (Applied Biosystems, Thermo Fisher Scientific Inc., Waltham, MA, USA). DNA sequences were checked by using Sequence Scanner Software 2 (Applied Biosystems, Thermo Fisher Scientific Inc., Waltham, MA, USA), and edited with VecScreen: Screen a Sequence for Vector Contamination on the National Center for Biotechnology Information website (https://www.ncbi.nlm.nih.gov/tools/vecscreen/). Subsequently, all sequences were analyzed for the presence of chimeras by using the Bellerophon 3 program [30] with default settings.

### 2.5. Statistical and Bioinformatic Analyses

Statistically significant differences among samples were tested using Analysis of Variance (ANOVA) and subsequently by using the “post-hoc” pairwise comparisons based on the Fisher’s least significant difference (LSD) procedure at the 95.0% confidence level, using the software Statgraphics x64 (Statpoint Technologies, Inc., Warrenton, VA, USA). Diversity indices were calculated from standardized profiles of individual rhizosphere samples, using the number and height of peaks in each profile as representations of the number and relative abundance of phylotypes. The Gini-Simpson index [31] was calculated as:

1 − *λ* = ∑ (*p_i_*^2^), (1)

where *λ* is Simpson diversity index and *p* is the proportion of an individual peak height relative to the sum of all peak heights. The Shannon’s diversity index [32] was calculated as:

*H’*= − ∑ (*p_i_*) (ln *p_i_*), (2)

and this index is commonly used to characterize species diversity in a community. The Pielou evenness index [33] was derived from Shannon’s diversity index and was calculated as:

*J’* = *H’*/*H’_max_*, (3)

where *H’_max_* = ln (*S*) and *S* represents the total number of species. Fungal communities in different samples were compared from T-RFLP profiles using height of fluorescence in individual T-RFs. These data were subsequently used for the Principal Component Analysis (PCA) using the scores of the first two principal components. Analysis of Similarities (ANOSIM) was used to determine if significant effects occurred among sewage sludge doses and time of sample collection (Two-way ANOSIM), and just among sewage sludge doses (One-way ANOSIM) with Euclidean distance measure. PCA and ANOSIM were evaluated by using the PAST (PAleontological STatistics) software version 3.19 [34]. The Basic Local Alignment Search Tool (BLAST) of the National Center for Biotechnology Information (NCBI) was used for searching of homologous sequences in the GenBank database. Phylogram based on DNA sequences was constructed by the UPGMA method using the MAFFT Multiple Sequence Alignment Software Version 7 [35].

### 2.6. GenBank Accession No.

DNA sequences of fungal clones from rhizosphere of control and sludge samples were deposited into the GenBank database under the accession numbers MH249155–MH249247 (PopSet 1476021752).

## 3. Results

### 3.1. Fungal Genetic Diversity

Fungal richness was evaluated as the first parameter and was higher in August in both years. In August 2014 richness was from 55 to 80 T-RFs and in November 2014 from 28 to 53 T-RFs. In August and December 2015 fungal richness was 30–76 and 24–34 T-RFs, respectively. Statistically significant differences were not detected among control, 5 t/ha and 15 t/ha samples in both years (ANOVA, α = 0.05). There were detected only temporal changes in fungal richness between two sampling times in each year (ANOVA, P = 0.0058 in 2014; P = 0.0008 in 2015). 

The fungal alpha diversity, i.e., values of genetic diversity indices (Gini-Simpson, Shannon and Evenness) had an upward trend between control and samples with SS only in August 2014 including statistical differences detected between them (Table 2). In other collection dates, the values showed a downward trend between control and samples with SS. Statistical differences among samples in individual collection date and diversity indices are shown in Table 2. 

### 3.2. T-RFLP and the Rhizosphere Fungal Communities

**Figure 1:**
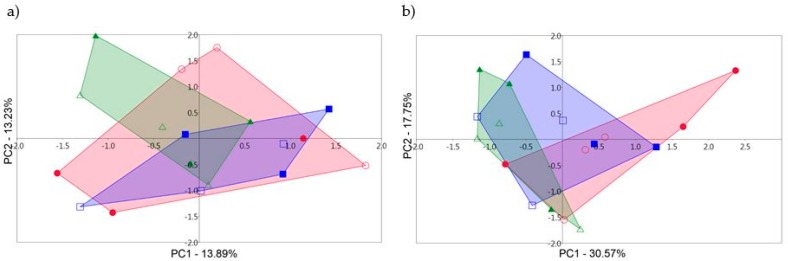
The principal component analysis (PCA) constructed from terminal-restriction fragment length polymorphism (T-RFLP) fluorescent data of fungal communities from *Arundo donax* rhizosphere collected in (**a**) 2014 and (**b**) 2015 in two sampling times each year in control samples and samples with sewage sludge (SS) in doses of 5 and 15 t/ha. PCA graphs explained a total of 27.12% and 48.32% (respectively) of the variability in the data. Filled symbols correspond to the samples collected in August 2014 and 2015; open symbols correspond to the samples collected in November 2014 and December 2015; red—control sample; blue—sample with 5 t/ha of SS; green—sample with 15 t/ha of SS.

The principal component analysis which was used to detect differences among fungal communities showed that control samples and samples with 5 t/ha and 15 t/ha were not grouped together in PCA graphs (Figure 1). Even control samples showed higher variability among themselves than samples with SS in 2014 (Figure 1a). This is less pronounced in 2015 (Figure 1b). Figure 1 shows that the areas belonging to individual sample variant overlap each other, so differences in fungal communities from control and samples with SS were insignificant. This finding was confirmed by ANOSIM which did not detect statistical differences among control and samples with SS and between collection dates in both years (Table 3). 

**Figure 2:**
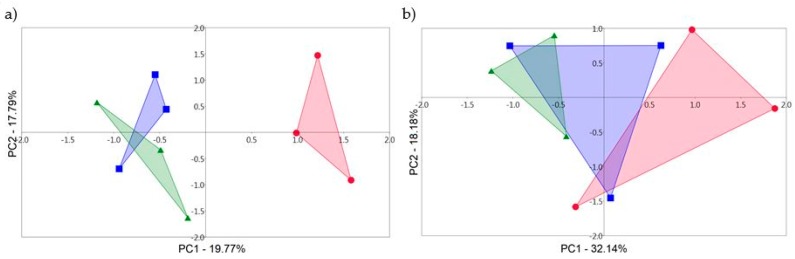
The principal component analysis (PCA) constructed from T-RFLP fluorescent data of fungal communities from *Arundo donax* rhizosphere collected in (**a**) 2014 and (**b**) 2015 in control samples and samples with sewage sludge (SS) in doses of 5 and 15 t/ha. PCA graphs explained a total of 37.56% and 50.32% (respectively) of the variability in the data. PCA was made by combining data from two sampling dates in each year into a single statistical table. Red—control sample; blue—sample with 5 t/ha of SS; green—sample with 15 t/ha of SS.

**Figure 3:**
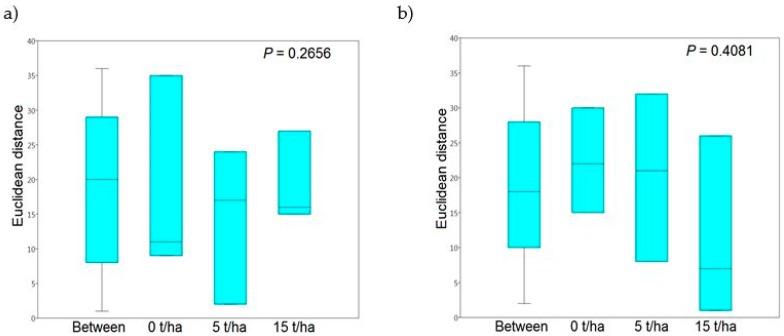
Box plots from One-way ANOSIM with Euclidean distance measure derived from the obtained data using principal component (PC) scores from principal component analysis in Figure 2 in (**a**) 2014 and (**b**) 2015 sampling years.

To determine whether sewage sludge alone has an effect on the genetic diversity of fungi in the *Arundo donax* rhizosphere, the samples were statistically evaluated so that the sampling dates in individual years were not taken into account. Principal component analysis suggests that there is an obvious difference between control samples and samples with SS in 2014 (Figure 2a). First principal component (PC) divided these samples and this distribution was maintained even when using with PC1 the remaining principal components (PC3–PC8, data not shown). On the other hand, in the use of PC2–PC8 principal components in various combinations, control samples with sludge samples were overlapped (data not shown). Such overlapping of samples, irrespective of the presence of sludge in the soil, was also observed in 2015 (Figure 2b). These results from PCA analyzes were also confirmed by ANOSIM using all eight principal components and statistically significant differences were not detected (P = 0.2656 in 2014 and P = 0.4081 in 2015; Figure 3).

### 3.3. 18S rDNA Sequencing and the Rhizosphere Fungal Communities

Fungal communities from the control sample and from sample with SS in dose of 15 t/ha collected in August 2014 were selected for constructing of 18S rDNA clone library. Each clone was analyzed by T-RFLP to select clones with a specific T-RFLP profile from a large number of isolated plasmids. Thirty-seven clones from the control sample and fifty-six clones from sample with SS in dose of 15 t/ha with specific T-RFLP profiles were sequenced and compared with 18S rDNA sequences of the closest fungal genera available in the GenBank database using Nucleotide BLAST (Table 4 and Table 5).

As shown in Table 4, comparison of our clone sequences with GenBank homologous sequences revealed that 32 clones (86.5%) from the control sample without SS belong to the arbuscular mycorrhizal fungi to the subphylum *Glomeromycotina* and the other 5 clones (13.5%) belong to the *Mortierellomycotina* (2 clones), *Ascomycota* (2 clones) and *Basidiomycota* (1 clone). Absolute (i.e., 100%) identity between compared DNA sequences was observed in 2 clones that related to the genus *Glomus*. Three genera of AM fungi were captured in the control sample—*Glomus*, *Funneliformis* and *Rhizophagus*. Their greatest homology to the compared sequences was 100%, 99% and 99%, respectively and *Glomus* species were represented at most times. The homology of our sequenced clones with AM fungi in the GenBank database was from 89% up, while 23 clones out of 32 were homologous above 97%. 

Table 5 shows that 39 clones (69.6%) belong to the AM fungi and *Glomeromycotina* group and 17 clones (30.4%) were outside this. Phylogenetic groups detected in addition to AM fungi were *Ascomycota* (11 clones), *Basidiomycota* (3 clones), *Mortierellomycotina* (1 clone) and 2 clones without further specification. Only 1 clone from all 56 sequenced showed 100% identity with GenBank sequence and it was a representative of *Glomus* sp. Other species from AM fungi detected in the sample with SS were *Funneliformis* and *Septoglomus*. Additionally, in this sludge sample *Glomus,* as a typical representative of AM fungi, was the most frequently represented. The homology of our clones with AM fungi in GenBank database was from 88% up and 24 clones from all 39 detected AM fungi were homologous above 97%.

**Figure 4:**
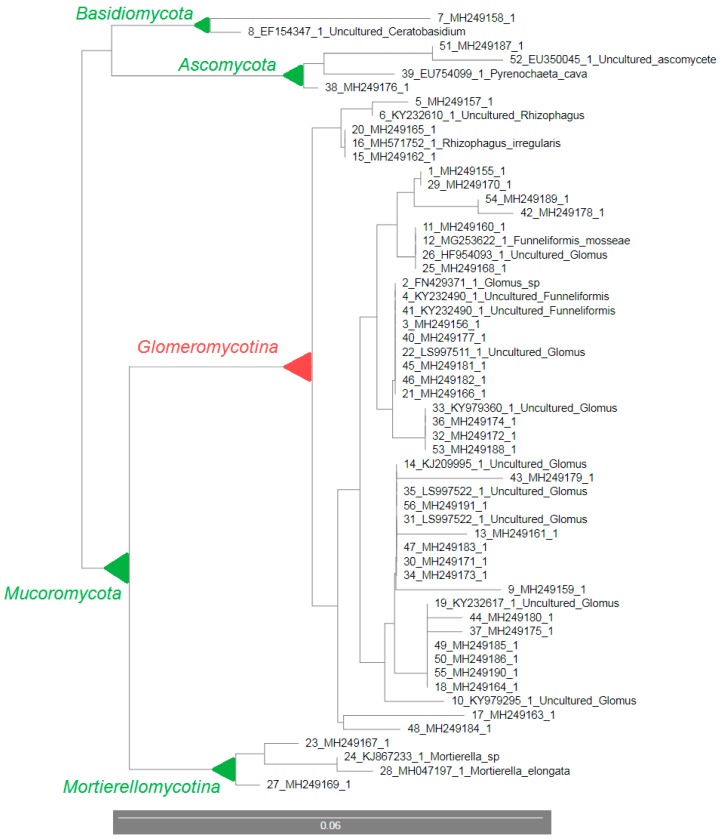
The phylogram constructed using Unweighted Neighbor-Joining method generated from the 18S rDNA sequences obtained from the rhizosphere of *Arundo donax* in the soil without sewage sludge (SS) with the sequences of fungi with the highest similarity (our sequences are indicated only with GenBank ID).

**Figure 5:**
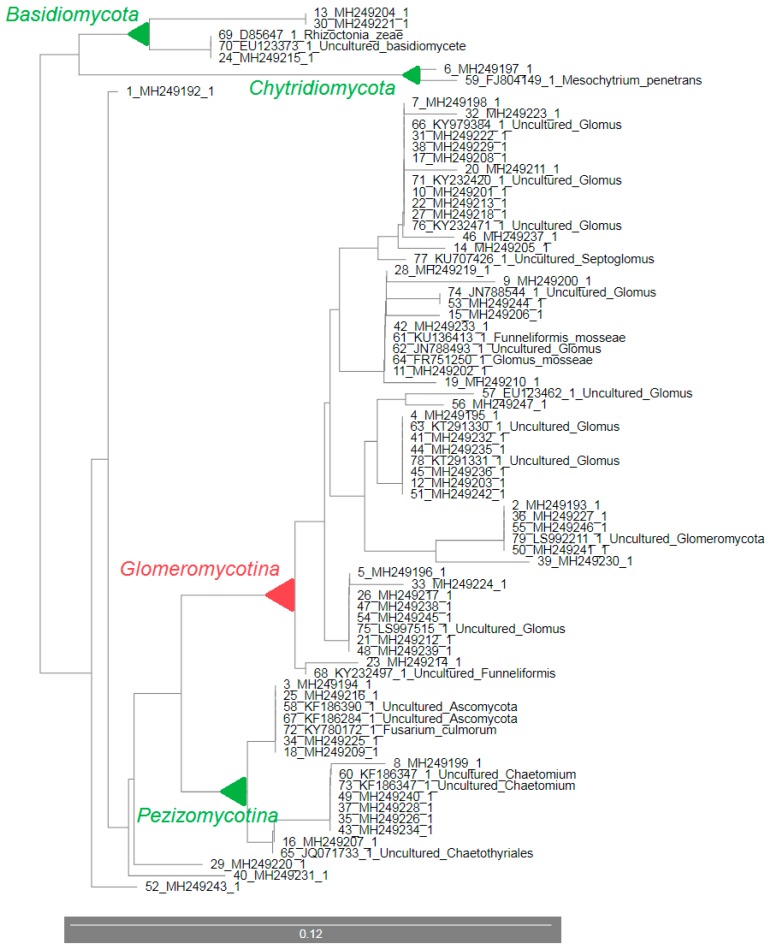
The phylogram constructed using Unweighted Neighbor-Joining method generated from the 18S rDNA sequences obtained from the rhizosphere of Arundo donax in the soil with 15 t/ha of sewage sludge (SS) with the sequences of fungi with the highest similarity (our sequences are indicated only with GenBank ID).

The phylograms constructed using 18S rDNA sequences from the control sample (Figure 4) and from the sample with 15 t/ha sewage sludge in the soil (Figure 5) confirmed the classification of these clones into individual phylogenetic groups as described above (Table 4 and Table 5) with little exceptions in the sample with SS. The largest group consisted of AM fungi, 86.5% in the *Arundo donax* rhizosphere in the control sample and 67.9% in the sample with SS in the soil. Higher diversity was observed in the sample with SS than in the control sample. At the same time, phylogram constructed from 18S rDNA fungal sequences from the sample with 15 t/ha SS included sequenced clones 2_3 (MH249194.1), 2_28 (MH249209.1), 2_35 (MH249216.1), and 2_46 (MH249225.1) to the subphylum *Pezizomycotina* (Figure 5) whilst in Table 5 these clones were classified only as *Ascomycota*. A similar case is with the clone 2_6 (MH249197.1). In Figure 5, this clone is assigned to the phylum *Chytridiomycota* while Table 5 shows it is without further specification. On the other hand, two clones 2_1 (MH249192.1) and 2_68 (MH249243.1) were classified otherwise without further classification in Figure 5 than in Table 5. Their greatest homology was with *Glomeromycetes* (95%) and *Ascomycota* (95%), respectively.

## 4. Discussion

This study was designed to examine the impact of sewage sludge from two municipal wastewater treatment plants (SS-A from Győr, Hungary and SS-B from Piešťany, Slovakia) on the fungal communities, with a greater focus on arbuscular mycorrhizal fungi in the rhizosphere of *Arundo donax* L. using the molecular T-RFLP method and 18S rDNA sequencing. The experiment was carried out for two consecutive years, 2014 and 2015, and the sludge was applied to the soil in concentrations of 5 t/ha and 15 t/ha. The addition of sewage sludge to the soil did not lead to changes in the fungal richness among control and samples with SS. Significant differences were observed only in collecting dates in both years with the higher richness in the summer period. Differences between 2014 and 2015 were in the alpha diversity (Table 2) which may have been caused by different sludge used in 2014 (SS-A) and 2015 (SS-B) (Table 1). The content of macroelements in both SSs was more similar than the content of microelements. The concentration of heavy metals in SS-A, as As, Cd, Cr, Cu, Ni, and Zn was 2.6, 2, 2.4, 2.9, 1.9, and 1.5 times higher than in SS-B, respectively. Only the concentration of Pb was 1.3 times higher in SS-B. This may be the reason for these differences in alpha diversity between 2014 and 2015. The results of diversity indices from 2014 are more similar to our assumption, which we made at the beginning of this research. The addition of sludge to the soil resulted in an increase in all three diversity indices in August 2014. The opposite trend was recorded in November 2014. There was a gradual, statistically significant decline in the diversity index values from control to sludge samples. In 2015, only one significant difference was detected in August between control and 15 t/ha samples. Our initial assumption was that the differences in alpha diversity would be more significant between control and sludge samples, but this was not confirmed in 2015. Although the used sludge contained heavy metals, their concentrations were well below the permitted limit under the Act No. 188/2003 in the Slovak Republic. Actually, the fungal communities are able to accept even greater concentrations of heavy metals in the soil [36,37], and changes in their communities are smaller than in the bacterial communities [38,39,40]. On the other hand, the opposite effect was observed by Mossa et al. [41] and Lin et al. [42]. They both found out that fungi were more sensitive than bacteria to heavy metal contaminations and simultaneously, Lin et al. [42] observed that *Ascomycota*, *Basidiomycota* and *Zygomycota* had strong tolerance to the heavy metals in the soil. Additionally, a ten-year sludge application with increasing concentrations of heavy metals (still within the upper limits accepted by the European Union) produced a significant decrease of total AMF spore numbers and the diversity of AMF populations in soil [43]. Concurrently, De Val et al. [43] have found that sensitive and relatively tolerant to high rates of heavy metals AMF ecotypes exist. It follows that AMF are able to tolerate a diverse range of metal concentrations, and inoculation with tolerant AMF into plant roots has huge potential to increase phytoaccumulation of heavy metals [44]. Even the high amounts of Cd added to the soil in the form of ash did not have a negative effect on the AMF mycorrhizal status [45].

Our results indicate that shift in the rhizosphere fungal communities between control and samples with SS, and between sampling times in both years based on PCA was not detected (Figure 1 and Figure 2). Even in 2014, greater variability in fungal communities was observed in control samples (Figure 1a). ANOSIM analysis confirmed these observations based on PCA and no statistically significant differences were detected between control and sludge samples or between sampling times in individual years (Table 3). In assessing the impact of SS alone on the rhizosphere fungal communities and neglecting the collection dates, PCA showed that in 2014 there is a significant shift in fungal communities. In Figure 2a control samples show a positive correlation and the sludge samples show a negative correlation within PC1 which determines the greatest degree of variability (19.77% in our case). These correlations of control and samples with SS were maintained using PC1 with other principal components. However, the use of the other principal components without PC1 showed that the differences in rhizosphere fungal communities between control and samples with SS disappeared. Although the use of other principal components is already with less variability, PC2 (17.79%) shows only slightly lower variability than PC1 and variability decreases slowly across other principal components (data not shown). ANOSIM confirmed these PCA assumptions and the fungal communities from the control samples did not show a significant shift compared to the fungal communities in the samples with SS (Figure 3). Our findings suggest that the short-term application of sewage sludge to the soil does not cause shift in the fungal communities in the *Arundo donax* rhizosphere. However, the long-term application of sewage sludge may produce opposite results. Mossa et al. [41] observed that more than 100 years of sewage sludge application to the soil has caused a shift in the fungal communities in the bulk soil and rhizosphere of “fodder maize”. They also found, by using T-RFLP, that Zn, unlike other heavy metals, can be considered as a good indicator of historical sewage sludge loading whereas its concentration of 700–1000 mg/kg appeared to be optimal for maximum microbial diversity (300 mg/kg of zinc is the maximum permitted concentration in the EU according to Council Directive 86/278/EEC). Tang et al. [46] in their review article assessed that the harmful effect of heavy metals on microorganisms depends on their availability and speciation rather than their concentration. Additionally, they reviewed that heavy metals after entering the soil can be affected by soil itself, thereby changing their toxicity [46]. Moreover, the sludge type, i.e., digested and undigested, appeared to have the greatest impact on the soil fungal community as sludge rich in heavy metals whose concentration exceeds the limits permitted by UK legislation [36].

Our results from 18S rDNA sequencing confirmed the previously known fact about NS31 and AM1 primers. These primers amplified, in addition to AMF, also non-AMF sequences to a small extent [28,29,47,48,49,50], like *Ascomycota* and *Basidiomycota* with a higher incidence of *Ascomycota* (14% from all sequences) in our case. However, many studies using these primers have significantly increased our knowledge about AMF communities in various environments [51,52,53,54,55,56]. In our case, more than 86% of the DNA sequences in the control sample and more than 69% in the sludge sample belonged to some group of AM fungi. The greatest homology of our sequences to those in the GenBank was with a representative of *Glomus* sp. (Table 4 and Table 5). Currently, there are around 60 well described *Glomus* species at www.amf-phylogeny.com [57] (last update 25 August 2019) and this number is not final. Additionally, the total number of AMF species described on this website, which is approximately 315 after this last update, is not final [57]. In addition to *Glomus* sp., *Funneliformis*, *Rhizophagus* and *Septoglomus* as representatives of AMF have also been found in the *Arundo donax* rhizosphere in control and sample with SS. AM fungi are microorganisms with very important and valuable functions in the ecosystem. Their potential for *Arundo donax* is studied and focused on enhancement of phytoremediation capabilities using arbuscular mycorrhiza [58], increase in photosynthesis and plant biomass accumulation [59], improvement of plant performance and productivity [60], or the acclimatization and establishment of *Arundo donax* plantlets [61,62]. However, the impact of sewage sludge on the AM fungal communities in the *Arundo donax* rhizosphere was not investigated yet. In our study, a greater spectrum of different fungal species was detected in the sample with SS than in the control (56 vs. 37 clones in the control) (Table 5, Figure 5). This is probably caused by the presence of sewage sludge in the soil which is a good source of valuable micro and macroelements as well as organic matter in high content [63,64]. These factors can create a suitable environment for the development and existence of various fungal taxa, other than AMF, which we were able to capture using primers NS31 and AM1. In addition, the presence of heavy metals in the SS, and subsequently in the soil, can promote and contribute to the presence of different taxa of soil fungi [42]. 

These presented results are from a two-year pot experiment, but it would be useful to investigate rhizospheric microorganisms under natural environmental conditions and for more consecutive years to further investigate the effect of sewage sludge. Furthermore, it would be beneficial also to investigate arbuscular mycorrhizal fungi directly from the plant roots to determine whether the sludge affects the mycorrhizal ability of these fungi.

## 5. Conclusions

The present study showed that short-term application of sewage sludge from municipal wastewater treatments plants in Hungary and Slovakia into the soil did not cause a shift in the overall fungal communities in the *Arundo donax* rhizosphere. Alpha diversity of fungal communities was more affected by SS-A than SS-B. This was probably due to the higher heavy metal content in this sludge although it was still well below the legal limit permitted in the EU. Sequencing of 18S rDNA showed that more various fungal taxa were detected in the sample with sewage sludge than in the control. At the same time, we confirmed that NS31 and AM1 primers that are used to amplify 18S rDNA from arbuscular mycorrhizal fungi amplified also fungi from *Ascomycota* and *Basidiomycota* to a lesser extent. However, from all detected fungi in the control and the sludge sample, *Glomus* sp. as a representative of arbuscular mycorrhizal fungi was the most represented. Furthermore, *Funneliformis* in both samples and *Rhizophagus* in control with *Septoglomus* in sludge samples were other representatives of AMF. 

## Figures and Tables

**Table 1:** Analysis of elements in used sewage sludge from Győr, Hungary (SS-A) and Piešťany, Slovakia (SS-B), and the conversion of heavy metal content to 1 kg of soil supplemented with sewage sludge in the dose of 5 t/ha and 15 t/ha.

**Macroelements**							
**Element**		**SS-A**		**SS-B**		**Unit (Method)**	
**N**		3.47		3.51		% (D)	
**P**		18,129.4		16,663.4		mg/kg (M)	
**K**		5958.6		2663.4		mg/kg (M)	
**Ca**		23,694.8		36,394.7		mg/kg (M)	
**Mg**		5704.7		6444.4		mg/kg (M)	
**Microelements/Heavy Metals**							
**Element**	**SS-A**	**SS-B**	**Unit (Method)**	**SS-A mg/kg Soil in 5 t/ha**	**SS-A mg/kg Soil in 15 t/ha**	**SS-B mg/kg Soil in 5 t/ha**	**SS-B mg/kg Soil in 15 t/ha**
**As**	8	3	mg/kg (RFS)	0.02	0.05	0.01	0.02
**Cd**	<2	<1	mg/kg (RFS)	<0.004	<0.01	<0.002	<0.01
**Cr**	84.7	36	mg/kg (RFS)	0.19	0.57	0.08	0.24
**Cu**	654	224	mg/kg (RFS)	1.47	4.40	0.50	1.51
**Ni**	42	22	mg/kg (RFS)	0.09	0.28	0.05	0.15
**Pb**	36	46	mg/kg (RFS)	0.08	0.24	0.10	0.31
**Zn**	1940	1269	mg/kg (RFS)	4.35	13.05	2.85	8.54

D—Dumas method, M—Mehlich III method, RFS—X-ray fluorescence spectrometry.

**Table 2:** Alpha diversity indices of fungal communities detected in the rhizosphere of *Arundo donax* in control and samples with sewage sludge (SS) in doses of 5 and 15 t/ha. The numbers behind the ± sign represent standard deviation (n = 12). The different letters denote statistically significant differences among samples evaluated, each sampling times separately (LSD, α = 0.05).

Year	Month	Dose of SS (t/ha)	1-λ Gini-Simpson	H Shannon	E_H_ Evenness
2014		0	0.9286 ± 0.0542 a	3.5022 ± 0.6170 a	0.7599 ± 0.0671 a
	August	5	0.9682 ± 0.0116 b	3.9654 ± 0.3958 b	0.8186 ± 0.0329 b
		15	0.9652 ± 0.0141 b	3.9435 ± 0.4920 b	0.8135 ± 0.0454 b
		0	0.9582 ± 0.0154 a	3.7126 ± 0.4006 a	0.7864 ± 0.0387 a
	November	5	0.9344 ± 0.0339 ab	3.2978 ± 0.6258 ab	0.7577 ± 0.0526 a
		15	0.9255 ± 0.0293 b	3.1817 ± 0.4973 b	0.7556 ± 0.0375 a
2015		0	0.8702 ± 0.1355 a	2.8653 ± 0.9912 a	0.7581 ± 0.1126 a
	August	5	0.8497 ± 0.1411 a	2.6768 ± 0.8520 a	0.7070 ± 0.1282 a
		15	0.8131 ± 0.0954 b	2.2390 ± 0.5040 a	0.6827 ± 0.0792 a
		0	0.8484 ± 0.0696 a	2.4569 ± 0.5780 a	0.7043 ± 0.1075 a
	December	5	0.8201 ± 0.0770 a	2.1926 ± 0.4296 a	0.7197 ± 0.1148 a
		15	0.8143 ± 0.0831 a	2.1938 ± 0.5422 a	0.7144 ± 0.0844 a

**Table 3:** The results of the Two-way ANOSIM derived from the obtained data using principal component (PC) scores from principal component analysis in Figure 1 in 2014 and 2015 sampling years and two collection dates in each year.

Similarity index	Euclidean distance	
Permutation N	9999	
	***P*-value**	
	**2014**	**2015**
Dose of SS	0.5485	0.1699
Collection date	0.2936	0.3465

**Table 4:** Comparison of sequenced clones from the rhizosphere of *Arundo donax* without SS in the soil with 18S rDNA sequences of fungi with the highest similarity using Nucleotide BLAST.

No.	Clone Name (GenBank ID)	GenBank ID with the Highest Similarity	GenBank Name	Similarity (%)	Phylogenetic Group
1	1_1 (MH249155.1)	FN429371.1	*Glomus* sp.	98	*Glomeraceae*
2	1_2 (MH249156.1)	KY232490.1	*Funneliformis*	99	*Glomeraceae*
3	1_3 (MH249157.1)	KY232610.1	*Rhizophagus*	99	*Glomeraceae*
4	1_4 (MH249158.1)	EF154347.1	*Ceratobasidium*	95	*Basidiomycota*
5	1_6 (MH249159.1)	KY979295.1	*Glomus*	98	*Glomeraceae*
6	1_8 (MH249160.1)	KY979397.1	*Glomus*	100	*Glomus*
7	1_20 (MH249161.1)	KJ209995.1	*Glomus*	97	*Glomeraceae*
8	1_24 (MH249162.1)	KY436352.1	*Rhizophagus irregularis*	95	*Glomeromycetes*
9	1_26 (MH249163.1)	KT291330.1	*Glomus*	99	*Glomeraceae*
10	1_27 (MH249164.1)	KY232617.1	*Glomus*	96	*Glomeromycetes*
11	1_28 (MH249165.1)	KX154254.1	*Rhizophagus*	99	*Glomeraceae*
12	1_30 (MH249166.1)	LN715052.1	*Glomus*	93	*Glomeromycetes*
13	1_31 (MH249167.1)	KJ867233.1	*Mortierella* sp.	99	*Mortierellomycotina*
14	1_33 (MH249168.1)	HF954093.1	*Glomus*	98	*Glomeraceae*
15	1_34 (MH249169.1)	MH047197.1	*Mortierella elongata*	98	*Mortierellomycotina*
16	1_35 (MH249170.1)	KY979409.1	*Glomus*	100	*Glomus*
17	1_39 (MH249171.1)	KY979298.1	*Glomus*	99	*Glomeraceae*
18	1_40 (MH249172.1)	KY979360.1	*Glomus*	97	*Glomeraceae*
19	1_42 (MH249173.1)	KY979298.1	*Glomus*	99	*Glomeraceae*
20	1_44 (MH249174.1)	KY979361.1	*Glomus*	99	*Glomeraceae*
21	1_45 (MH249175.1)	KY232615.1	*Glomus*	97	*Glomeraceae*
22	1_47 (MH249176.1)	EU754099.1	*Pyrenochaeta cava*	86	*Ascomycota*
23	1_48 (MH249177.1)	KY232490.1	*Funneliformis*	98	*Glomeraceae*
24	1_49 (MH249178.1)	KY979384.1	*Glomus*	91	*Glomeromycetes*
25	1_50 (MH249179.1)	GU353937.1	*Glomus*	89	*Glomeromycetes*
26	1_51 (MH249180.1)	KY979297.1	*Glomus*	98	*Glomeraceae*
27	1_53 (MH249181.1)	LN715041.1	*Glomus*	99	*Glomeraceae*
28	1_55 (MH249182.1)	KY232490.1	*Funneliformis*	97	*Glomeraceae*
29	1_56 (MH249183.1)	KC797120.1	*Glomus*	98	*Glomeraceae*
30	1_58 (MH249184.1)	AB695021.1	*Glomus*	95	*Glomeromycetes*
31	1_62 (MH249185.1)	KY232617.1	*Glomus*	99	*Glomeraceae*
32	1_66 (MH249186.1)	JQ218167.1	*Glomus*	96	*Glomeromycetes*
33	1_67 (MH249187.1)	EU350045.1	*Ascomycota*	97	*Ascomycota*
34	1_68 (MH249188.1)	KY979360.1	*Glomus*	90	*Glomeromycetes*
35	1_69 (MH249189.1)	KY232454.1	*Glomus*	99	*Glomeraceae*
36	1_71 (MH249190.1)	JQ218180.1	*Glomus*	92	*Glomeromycetes*
37	1_72 (MH249191.1)	KY979298.1	*Glomus*	97	*Glomeraceae*

**Table 5:** Comparison of sequenced clones from the rhizosphere of *Arundo donax* with 15 t/ha of SS in the soil with 18S rDNA sequences of fungi with the highest similarity using Nucleotide BLAST.

No.	Clone Name (GenBank ID)	GenBank ID with the Highest Similarity	GenBank Name	Similarity (%)	Phylogenetic Group
1	2_1 (MH249192.1)	EU123462.1	*Glomus*	95	*Glomeromycetes*
2	2_2 (MH249193.1)	KT291279.1	*Glomus*	98	*Glomeraceae*
3	2_3 (MH249194.1)	EU350045.1	*Ascomycota*	99	*Ascomycota*
4	2_4 (MH249195.1)	KT291330.1	*Glomus*	99	*Glomeraceae*
5	2_5 (MH249196.1)	AB695021.1	*Glomus*	99	*Glomeraceae*
6	2_6 (MH249197.1)	JF972676.1	*Eukaryote*	99	*Eukaryota*
7	2_7 (MH249198.1)	KY232471.1	*Glomus*	99	*Glomeraceae*
8	2_8 (MH249199.1)	KF186347.1	*Chaetomium*	99	*Ascomycota*
9	2_10 (MH249200.1)	KU136413.1	*Funneliformis mosseae*	90	*Glomeromycetes*
10	2_17 (MH249201.1)	KY232454.1	*Glomus*	94	*Glomeromycetes*
11	2_18 (MH249202.1)	JN788493.1	*Glomus*	94	*Glomeromycetes*
12	2_20 (MH249203.1)	KT291330.1	*Glomus*	96	*Glomeromycetes*
13	2_21 (MH249204.1)	EU622843.1	*Laetisaria arvalis*	99	*Basidiomycota*
14	2_22 (MH249205.1)	KJ209912.1	*Glomus*	93	*Glomeromycetes*
15	2_23 (MH249206.1)	FR751250.1	*Glomus mosseae*	88	*Glomeromycetes*
16	2_24 (MH249207.1)	JQ071733.1	*Chaetothyriales*	96	*Ascomycota*
17	2_25 (MH249208.1)	KY979384.1	*Glomus*	99	*Glomeraceae*
18	2_28 (MH249209.1)	EU123341.1	*Ascomycota*	95	*Ascomycota*
19	2_29 (MH249210.1)	LN715042.1	*Funneliformis*	93	*Glomeromycetes*
20	2_30 (MH249211.1)	KY979384.1	*Glomus*	98	*Glomeraceae*
21	2_31 (MH249212.1)	KM602163.1	*Glomeromycota*	96	*Glomeromycota*
22	2_32 (MH249213.1)	KY979384.1	*Glomus*	99	*Glomeraceae*
23	2_33 (MH249214.1)	KY232497.1	*Funneliformis*	99	*Glomeraceae*
24	2_34 (MH249215.1)	EU123373.1	*Basidiomycota*	96	*Basidiomycota*
25	2_35 (MH249216.1)	EU350045.1	*Ascomycota*	98	*Ascomycota*
26	2_36 (MH249217.1)	KY979355.1	*Glomus*	98	*Glomeraceae*
27	2_37 (MH249218.1)	KY232438.1	*Glomus*	99	*Glomeraceae*
28	2_39 (MH249219.1)	JN788427.1	*Glomus*	98	*Glomeraceae*
29	2_40 (MH249220.1)	MH047197.1	*Mortierella elongata*	93	*Mortierellomycotina*
30	2_41 (MH249221.1)	EU622843.1	*Laetisaria arvalis*	93	*Basidiomycota*
31	2_42 (MH249222.1)	KY979376.1	*Glomus*	99	*Glomeraceae*
32	2_43 (MH249223.1)	KY979386.1	*Glomus*	94	*Glomeromycetes*
33	2_44 (MH249224.1)	AB698615.1	*Glomus*	88	*Glomeromycetes*
34	2_46 (MH249225.1)	EU123343.1	*Ascomycota*	97	*Ascomycota*
35	2_48 (MH249226.1)	KF186347.1	*Chaetomium*	96	*Ascomycota*
36	2_52 (MH249227.1)	KY979298.1	*Glomus*	99	*Glomeraceae*
37	2_53 (MH249228.1)	KF186347.1	*Chaetomium*	99	*Ascomycota*
38	2_54 (MH249229.1)	KU707426.1	*Septoglomus*	92	*Glomeraceae*
39	2_55 (MH249230.1)	KY232615.1	*Glomus*	99	*Glomeraceae*
40	2_56 (MH249231.1)	AB534478.1	Uncultured fungus	96	*Fungi*
41	2_57 (MH249232.1)	KT291330.1	*Glomus*	99	*Glomeraceae*
42	2_58 (MH249233.1)	KY979398.1	*Glomus*	99	*Glomeraceae*
43	2_59 (MH249234.1)	KF186347.1	*Chaetomium*	93	*Ascomycota*
44	2_60 (MH249235.1)	KY232519.1	*Glomus*	99	*Glomeraceae*
45	2_61 (MH249236.1)	KY232519.1	*Glomus*	99	*Glomeraceae*
46	2_62 (MH249237.1)	KY232454.1	*Glomus*	99	*Glomeraceae*
47	2_63 (MH249238.1)	KY979355.1	*Glomus*	99	*Glomeraceae*
48	2_64 (MH249239.1)	KY979355.1	*Glomus*	96	*Glomeraceae*
49	2_65 (MH249240.1)	KF186347.1	*Chaetomium*	99	*Ascomycota*
50	2_66 (MH249241.1)	KY979298.1	*Glomus*	99	*Glomeraceae*
51	2_67 (MH249242.1)	KY232519.1	*Glomus*	93	*Glomeromycetes*
52	2_68 (MH249243.1)	AF202291.1	*Labyrinthomyces* sp.	95	*Ascomycota*
53	2_69 (MH249244.1)	JN788544.1	*Glomus*	99	*Glomeraceae*
54	2_70 (MH249245.1)	KY979355.1	*Glomus*	99	*Glomeraceae*
55	2_71 (MH249246.1)	KY979298.1	*Glomus*	100	*Glomus*
56	2_72 (MH249247.1)	EF041057.1	*Glomus*	92	*Glomeromycetes*
